# Does health literacy moderate the psychological pathways of physical activity from guideline awareness to behavior? A multi-group structural equation modeling

**DOI:** 10.1186/s12889-023-15012-3

**Published:** 2023-01-14

**Authors:** Takayuki Tajima, Kazuhiro Harada, Yuko Oguma, Susumu S. Sawada

**Affiliations:** 1 Graduate School of Human Health Sciences, Tokyo Metropolitan University, 7-2-10 Higashiogu, Arakawa-Ku, Tokyo, 116-8551 Japan; 2 Sports Medicine Research Center, Keio University, 4-1-1 Hiyoshi, Kohoku, Yokohama, Kanagawa 223-8521 Japan; 3 Graduate School of Human Development and Environment, Kobe University, 3-11, Tsurukabuto, Kobe, Nada 657-8501 Japan; 4 Graduate School of Health Management, Keio University, 4411 Endo, Fujisawa, Kanagawa 252-0883 Japan; 5 Faculty of Sport Sciences, Waseda University, 2-579-15, Mikajima, Tokorozawa, Saitama 359-1192 Japan

**Keywords:** Guideline, Awareness, Knowledge, Attitude, Intention, Exercise, Health literacy, Surveys and Questionnaires, Latent Class Analysis

## Abstract

**Background:**

Awareness, knowledge, beliefs, and behavioral intentions of physical activity (PA) guidelines may be important mediating factors for promoting PA. However, these pathways of the psychological process to PA behavior have not been examined. These pathways may differ depending on health literacy levels. This study investigated the pathways to PA, from guideline awareness to behavior, and further examined whether they differed by health literacy.

**Methods:**

A cross-sectional study was conducted with 7,000 Japanese participants aged 20–69 years. The participants were registered with an Internet survey company. Participants’ awareness, knowledge, beliefs, and behavioral intentions regarding the PA guidelines of Japan, the volume of moderate-to-vigorous intensity PA, activity level, and health literacy were examined through a questionnaire. The PA pathways, from guideline awareness to behavior, were examined by structural equation modeling (SEM), with PA behavior as the dependent variable. Multi-group SEM was conducted to examine the moderating effect of health literacy on PA pathways. Health literacy scores were dichotomized into high and low groups in multi-group modeling by the median split.

**Results:**

SEM revealed that PA guideline awareness directly affects PA behavior and has certain indirect effects through the mediation of knowledge, beliefs, and behavioral intentions. Furthermore, the multi-group SEM showed that the proportion of indirect effects (path coefficient [PC]: 0.11, 95% confidence interval [CI]: 0.10–0.13) was higher than direct effects (PC: 0.07, 95%CI: 0.03–0.11) in the high-health literacy group. In contrast, the proportion of direct effects (PC: 0.22, 95%CI: 0.15–0.30) was higher than indirect effects (PC: 0.06, 95%CI: 0.05–0.07) in the low-health literacy group.

**Conclusions:**

PA guideline awareness is both directly and indirectly associated with PA behavior, mediated by psychological pathways of knowledge, beliefs, and behavioral intentions, and influenced by health literacy. These results suggest that health literacy should be considered when implementing PA guideline-based interventions.

**Supplementary Information:**

The online version contains supplementary material available at 10.1186/s12889-023-15012-3.

## Background

Physical activity (PA) is an important factor to maintain and improve health in adults, including reductions in all-cause and cardiovascular mortality, development of type 2 diabetes and certain cancers, and improvements in mental health [1–6]. However, physical inactivity has become a global public health concern [7] that needs to be addressed. PA guidelines have been developed in many countries based on epidemiological findings to promote PA among the general population [1–6]. These guidelines provide recommendations on PA intensity, frequency, duration, and types of activities. For example, WHO guidelines established in 2020 recommended adults perform at least 150–300 min or 75–150 min per week of moderate-intensity aerobic PA or vigorous-intensity PA, respectively [1]. In Japan, the Ministry of Health, Labor and Welfare developed PA guidelines in 2013 [6]. These guidelines recommend 60 min of daily PA of at least 3 metabolic equivalents (METs) for those between 18–64 years of age, and 40 min of daily PA of any intensity for those aged 65 years or older. The Japanese PA guidelines differ from the recommendations of other countries, including those of the WHO. The reasons are: 1) a subanalysis of a comprehensive meta-analysis showed that having a PA of more than 22.5 METs-hours per week had a risk reduction effect on non-communicable diseases and functional decline in the Japanese population, and 2) the average amount of PA in the Japanese population was higher than that in the international recommendations [6]. In addition, Japanese PA guidelines also includes the key message “Plus-Ten (be active for 10 more minutes than now)” as a challenge that everyone should implement in their daily lives [8]. A meta-analysis suggests that an increase of 10 min of daily PA reduces the risk of non-communicable diseases and functional decline by 3.2% in Japanese populations [6]. Furthermore, the benefit of further increasing PA has been shown in people who have already reached the recommended daily time committed to PA [6]. Therefore, the "Plus-ten" is used as an important message for all populations.

These guidelines not only provide detailed information for professionals but also contain “summary sheets” that explain the guidelines for ease of understanding of the public. In Japan, “Active Guide” has been developed as a “summary sheet” for the public in 2013 [9]. The purpose of this guide is to communicate the significance of PA and encourage PA commitment to a broader public. Furthermore, the guide is structured to emphasize "Plus-ten" as a message that is easy to understand and attractive to the public and contains a lot of information to promote behavioral changes, such as awareness of the environment, opportunities to perform PA, and the use of the support provided by other people. Therefore, promoting the Active Guide to the public and increasing its awareness may encourage PA among Japanese people.

Several factors have been suggested to promote PA, including awareness and knowledge, beliefs about, and behavioral intentions regarding PA guidelines [10, 11]. For example, Cavil & Bauman suggested a logic model for promoting PA [11]. The first step in this model is to make the participants aware of the PA guidelines. It is hypothesized that awareness leads to obtaining knowledge of the recommended amounts of PA and specific messages of the guidelines, which in turn leads to changes in beliefs and attitudes about physical activity, which brings on behavioral intentions, and finally behavioral change. As indicated in this model, understanding the pathways of PA, the psychological processes that begin with awareness of guidelines and leads to behavior change, can provide insights to improving the efficiency of PA promotion. Various interventions, such as mass media campaigns and dietary guidelines, have examined the pathways from awareness to behavior [12–15]. These awareness campaigns showed a hierarchical effect mediated by knowledge, beliefs, and behavioral intention, which led to behavior. This framework could also be applied to examine the PA pathway from guideline awareness to behavior. Our previous studies and that conducted in other countries have shown associations between awareness or knowledge of PA guidelines and the amount of PA [16–24]. However, it remains unclear whether these associations are mediated by psychological pathways of belief and behavioral intention.

In addition, these pathways may be influenced by health literacy. As such, the effectiveness or issues of the guidelines as a tool for behavior change may also be impacted by health literacy.

There are various concepts and definitions of health literacy. In the health promotion domain, there are three classifications: functional health literacy, interactional health literacy, and critical health literacy [25]. Reading comprehension skills (functional health literacy) are essential for effective health education. However, this ability alone does not necessarily translate into action. The ability to communicate well with people around them in a supportive environment (interactional health literacy) and critically analyze information and use it to gain more control over a situation (critical health literacy) are also required. Furthermore, a comprehensive health literacy conceptual framework was proposed by Sørensen et al. [26]. They defined health literacy as the knowledge, motivation, and competence to access, understand, evaluate, and apply health information in making decisions about health promotion [26]. According to these concepts and definitions, people with high health literacy are more likely to have access to information on PA guidelines than those with low health literacy. As a consequence, their understanding and application of the guidelines may promote PA. Recent reviews of health literacy and PA level have also shown that high health literacy is associated with high PA level [27]. However, few studies have focused on the impact of health literacy on the PA pathway from guideline awareness to behavior. In our previous study, people with higher health literacy were more aware of PA guidelines. However, the positive association between PA guideline awareness and PA level was more pronounced in people with low health literacy than in those with high health literacy [19]. This finding suggests that the PA pathway from guideline awareness to practice may be influenced by health literacy.

Therefore, the research questions for this cross-sectional study are as follows: (1) whether the PA guideline awareness was associated with PA behavior, mediated by the psychological pathways of knowledge, beliefs, and behavioral intentions; and (2) whether these psychological pathways were influenced by health literacy.

## Methods

### Participants and data collection

In this cross-sectional study, an online survey was conducted between October and November 2020. The target population included registered users of a social survey company (approximately 1.07 million users as of October 2020) between the ages of 20 and 69 years. The users registered with this company are an open-access panel recruited in various ways that does not rely on a specific platform. Among these users, 7,000 responses were requested to be collected. The sample size was determined to be as large as possible based on available funding resources. The registered participants, stratified by age group (e.g., 20–29, 30–39 years), sex (men/women), and education (junior high school/ high school/ 2-year college or vocational school/ 4-year college or more), were randomly selected so that the study population would match the population distribution in Japan. The survey company sent an e-mail to 23,188 registered users with the details of the study and a link to the response web page. The survey was closed when the number of respondents reached the target number in each stratum and when the total number of respondents reached 7,000.

This study was conducted after obtaining approval from the ethical review committees of Arakawa campus, Tokyo Metropolitan University (No. 20039), and Graduate School of Human Development and Environment, Kobe University (No. 455). All methods were carried out in accordance with the Japanese ethical guidelines for medical and biological research involving human subjects and the ethical standards of the Declaration of Helsinki. Consent for this study was obtained by presenting an explanatory statement at the beginning and requesting that only those who fully understood and consented to the statement would access the survey site. This survey has multiple research objectives and two papers have already been published using this survey [19, 23]. The first paper examined how each construct of the PA guidelines (awareness, knowledge, beliefs, and behavioral intentions), which consists of a logic model, was related to PA and sedentary behavior [23]. The second paper examined the relationship between health literacy, awareness of the PA guidelines, and physical activity levels [19]. Based on these results, the novelty of this present study is that it examined the psychological pathways from awareness of PA guidelines to PA behaviors, considering health literacy levels.

### Measures

#### Participants’ characteristics

Based on previous studies, we chose participant characteristics associated with guideline awareness and knowledge, PA behavior, and health literacy [16–24]. Classification of educational background and household income was based on the information in other studies and surveys in Japan [19, 23, 28]. The characteristics of the participants were as follows: age, sex, current marital status, current working status, educational background (junior high school /high school /2-year college or vocational school /4-year college or more), and household income level (< \ 2 million / < \ 4 million / < \ 6 million / > \ 6 million).

#### Awareness of PA guidelines

Awareness of the Japanese PA guidelines was surveyed using the unprompted and prompted recalls. The unprompted recall method does not present clues, such as options or pictures, and the participant is asked to respond freely based on their memory [29]. For this recall, the participants were asked whether they had heard of the Japanese PA guidelines. Those who answered “yes” to this question were asked to freely provide the name of the guidelines. The first and second authors judged these free responses as correct, classified as “aware,” or incorrect. Prompted recall is a method to evoke the participants' memory by presenting hints, such as options or pictures, and asking them to respond [18, 22, 30]. For this recall, two types of recall were used: written and illustrated recalls. In the written recall, we investigated the awareness of the Active Guide and “Plus-ten.” Participants were asked whether they had ever heard of these two terms. Among four options, including “I know the contents,” “I have heard of it but do not know the contents,” “I have never heard of it,” and “I learned it for the first time in this survey,” those who answered, “I know the contents” or “I have heard of it but do not know the contents” were classified as “aware” [18, 30]. In the illustrated recall, we presented an illustration of the Active Guide and asked the participants to respond either “yes” or “no” if they had ever seen or heard of a campaign that encouraged them to exercise or move their bodies. Those who answered “yes” were classified as “aware” [22]. Finally, we defined those who recognized any of the recall as “aware” of the PA guidelines.

#### Knowledge of PA guidelines

Next, we assessed knowledge of the PA guidelines. While awareness of the guidelines assessed whether participants had seen or recognized the guidelines, knowledge investigated whether they understood the recommended quantities, which are an essential component of the guidelines. Knowledge of the PA guidelines was based on numerical responses with open-text fields to the following three items: (1) recommended PA amount for individuals aged 18–64 years, (2) recommended amount of physical activity for individuals aged 65 years or older, and (3) PA amount that should currently be increased. For each item, 60 min/day, 40 min/day, and 10 min/day were considered correct answers. Among respondents aged 18–64 years, those who correctly answered both items (1) and (3) were classified as “adequate,” and those who incorrectly answered item (1) and/or (3) were classified as “inadequate.” Similarly, among respondents aged 65 years and older, those who correctly answered both items (2) and (3) were classified as “adequate”, and those who incorrectly answered item (2) and/or (3) were classified as “inadequate.”

#### Beliefs about PA guidelines

Eight items, which were developed based on the information in the Active Guide, were used to assess beliefs about PA guidelines (e.g., I think increasing the amount of time spent on physical activity, even if only a little, helps improve my health). The beliefs included items related to PA and health promotion and the development of PA habits (Additional file 1). The fitness of the model was confirmed by confirmatory factor analysis [23]. The goodness of fit of the model was root mean square error of approximation (RMSEA) = 0.070, comparative fit of index (CFI) = 0.985, and Tucker-Levis index (TLI) = 0.979, all of which were generally acceptable fit indices. The test–retest reliability conducted 2 weeks later was r = 0.45. Each item was surveyed using a 5-point scale. The calculated total score after each item ranged from 1 (completely disagree) to 5 (very strongly agree).

#### Behavioral intentions regarding PA participation according to recommended guidelines

For behavioral intentions, two items were developed: (1) “Do you intend to move your body for 60 min (18–64 years)/ 40 min (65 years and older) per day?” and (2) “Do you intend to move your body 10 min more than you do currently?” The options for both items were on a five-point scale from 1 (not at all) to 5 (very strongly). The average of the scores of the two items was calculated and used to score behavioral intention.

#### PA

PA was assessed using two questionnaires used in the Japan Public Health Center-based prospective Study (JPHC Study) [31] and in specific medical checkups and health guidance [32]. As the Active Guide prioritizes the message “Plus-ten” rather than the recommended levels of PA, the present study did not dichotomize the PA behaviors by meeting the recommended level. From the questionnaire of the JPHC study, moderate-to-vigorous-intensity PA (MVPA) was calculated using the method reported by Kikuchi et al. [33]. This questionnaire calculates MVPA using two items from occupation/household activities (walking/strenuous work) and three items from leisure time activities (walking fast/ light to moderate exercise such as golf, gardening/ vigorous exercise such as tennis, jogging, aerobics, and swimming) [31, 33]. Compared to 24-h activity records, the questionnaire showed moderate correlation (rho = 0.672) with MVPA, and moderate reliability (rho = 0.645) with retests conducted 3–6 months after the survey [33]. From the questionnaire of the specific medical checkups and health guidance, activity level was calculated using three items related to physical activity (exercise, daily physical activity, and walking speed). The participants were asked about their exercise, PA, and walking speed as follows: (1) “Do you engage in light sweaty exercise for at least 30 min for at least 2 days a week for at least 1 year?”, (2) “Do you engage in walking or similar PA for at least 1 h/day in your daily life”, and (3) “Do you walk faster than your peers of about the same age?”. The participants answered each question with a “yes” or “no,” and the activity level was classified into two groups: low (the number of “yes” responses was 0 or 1) and high (the number of “yes” responses was 2 or 3). It is because Kawakami et al. showed that this stratification approach yielded the highest discriminant validity (73% sensitivity and 68% specificity) to detect a recommended level of accelerometer-measured PA [32].

#### Health literacy

For health literacy, we used the Japanese version of the Communicative and Critical Health Literacy (CCHL) [34]. The CCHL consists of three assessment items of interactive health literacy (e.g. seeking information from various sources) and two assessment items of critical health literacy (e.g. considering the credibility of the information). Each item was answered using a five-point scale ranging from “Not at all” (1 point) to “Strongly agree” (5 points). The average score of the five items was used as the scale score (1–5 points). The internal consistency (Cronbach’s alpha) was 0.86 [34]. Construct validity of this scale was confirmed by examining its associations with health behaviors, coping styles, and somatic symptoms [34].

#### Order of measures

To minimize the influence of each survey item on the others, the following order was used for the survey: awareness of the Active Guide (unprompted recall), awareness of the Active Guide (written prompted recall), knowledge of the Active Guide, awareness of “Plus-ten” (written prompted recall), awareness of the Active Guide (illustrated prompted recall), behavioral intentions, and beliefs. Each question was displayed on a separate web screen. Additionally, it was not possible to return to the previous question.

### Statistical analysis

#### Pathways from awareness of the guidelines to physical activity behavior among all respondents

The present study conducted the structural equation modeling to examine the PA pathways from the guideline awareness to behavior. The dependent variable was PA behavior. PA behavior was treated as a latent variable, defined by two observed variables: volume of MVPA (METs-hours per day) measured by the JPHC Study’s questionnaire [31, 33] and sufficient PA level (insufficient = 0, sufficient = 1) measured by Kawakami et al. [32]. PA beliefs and behavioral intention were also treated as latent variables from corresponding items. Awareness (not aware = 0, aware = 1) and knowledge (inadequate = 0, adequate = 1) of PA guidelines were treated as observed variables.

**Fig. 1:**
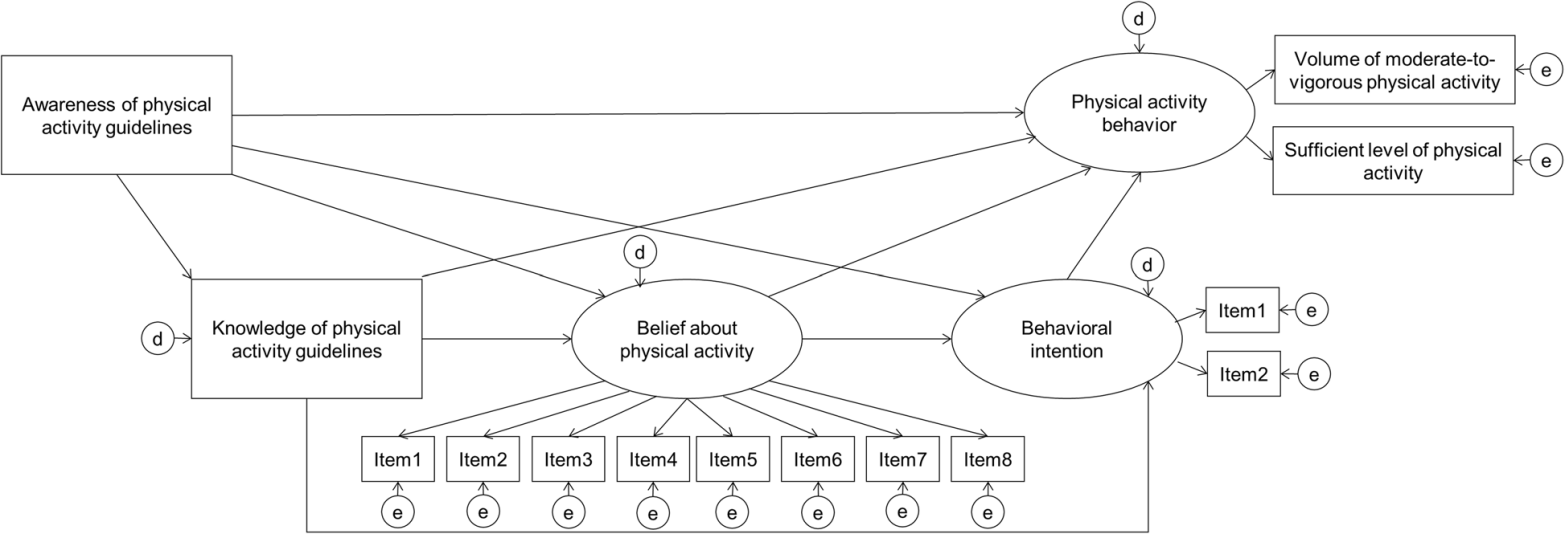
Hypothesized models for pathways from PA guideline awareness to behavior. Awareness (not aware = 0, aware = 1) and knowledge (inadequate = 0, adequate = 1) of PA guidelines and PA amount (insufficient = 0, sufficient = 1) were dummy variables.

In the initial model, the present study examined the statistical significances of the following 10 standardized path coefficients based on the logic model: path from awareness to knowledge, belief, behavioral intention, and behavior; path from knowledge to belief, behavioral intention, and behavior; path from belief to behavioral intention and behavior, and path from behavioral intention to behavior (Fig. 1). Subsequently, we revised the model by removing insignificant paths. Chi-squared, CFI, TLI, RMSEA, and AIC values were evaluated as the model fit indices. The cutoff points for CFI/TLI and RMSEA were set as 0.95 and 0.06, respectively [35]. Using the bias-corrected bootstrap method (5,000 bootstrap samples), the present study estimated standardized direct, indirect, and total effects and 95% confidence intervals of awareness and knowledge of, beliefs about, and behavioral intention regarding PA behavior in the revised model. If the total and indirect effects of awareness on PA behavior were positive and statistically significant, the present study confirmed that there were mediating effects of this psychological pathway on PA behavior, which corresponded to the first research question.

#### Moderating effects of health literacy on PA pathways from guideline awareness to behavior

Multi-group structural equation modeling was performed to examine the moderating effects of health literacy on the PA pathways from guideline awareness to behavior. The health literacy scores were dichotomized into low or high groups by the median split with the score of 3.40. The models were compared between low- and high-health literacy groups.

The present study implemented the multi-group structural equation modeling in three steps. In the first step, the unconstrained model was developed. The unconstrained model did not have any equality constraints for all parameters between the low- and high-health literacy groups. In the second step, we examined the model fit indices of the models constraining the following parameters between the low and high health literacy groups as equal: the path coefficients and variance within each latent variable (beliefs, behavioral intention, and PA behavior), variances of five main variables (observed variable of awareness, observed variable of knowledge, latent variable of beliefs, latent variable of behavioral intention, and latent variable of PA behavior), and path coefficients among main variables. For each parameter, a significant change in Chi-squared in the constrained model indicated that the corresponding parameter was different between the low- and high-health literacy groups. An insignificant change in Chi-squared in the constrained model indicated that equality constraint of the corresponding parameter between the two groups was reasonable. Thus, if the changes in Chi-squared were significant for the coefficients of the paths from awareness to knowledge, beliefs, intention, or PA behavior, the present study confirmed the moderating effects of health literacy on these psychological pathways, which addresses the second research goal. In the third step, the final model was constructed. The present study added the equality constraints identified in the second step on the unconstrained model. Using the bias-corrected bootstrap method (5,000 bootstrap samples), standardized direct, indirect, and total effects and 95% confidence intervals of awareness and knowledge of, beliefs about, and behavioral intention regarding PA behavior were estimated for the low- and high-health literacy groups.

Statistical significance was set at *p* < 0.05. Due to the nature of the web-based questionnaire survey, the database did not have missing values. The present study used AMOS v.25.0 (IBM Japan, Ltd., Tokyo, Japan) to perform the structural equation modeling.

## Results

### Participant characteristics

Table 1 shows the characteristics of respondents. Awareness of the Active Guide was 1.7% for unprompted recall. For awareness in written recall for prompted recall, the Active Guide was 13.4%, and “Plus-ten” was 6.8%. In the illustrated recall, awareness of the Active Guide was 5.3%. A total of 15.1% of the participants were aware of the Active Guide in any of the recalls. The correct response rate for the knowledge of the Active Guide was 37.2% for "(1) recommended PA amount for individuals aged 18–64 years," 7.0% for "(2) recommended amount of physical activity for individuals aged 65 years or older," and 24.8% for "(3) PA amount that should currently be increased." Finally, 6.6% of the participants correctly responded to all three items for knowledge. The average score of items for belief about PA were 3.5 (0.8) [mean (SD)]. The average score of items for behavioral intention were 2.7 (1.2) regarding the recommended amount of Active Guide, and 3.0 (1.2) regarding "Plus-ten”. Volume of moderate-to-vigorous PA was 10.4 (12.4) METs-hours per day. Sufficient level of PA was 31.0%.

**Table 1:** Characteristics of respondents

	M (SD) or %
Age (years) M(SD)	46.2 (13.4)
Sex, %	
Men	50.4%
Women	49.6%
Current marital status, %	
No	46.6%
Yes	53.4%
Current working status, %	
No	33.6%
Yes	66.4%
Educational background, %	
4-year college or more	27.2%
2-year college or vocational school	21.1%
High school	44.8%
Junior high school	6.9%
Household income level, %	
> \ 6 million	33.6%
< \ 6 million	24.1%
< \ 4 million	26.7%
< \ 2 million	15.6%
Health literacy score (range, 1 to 5), M (SD)	3.4 (0.7)
Awareness of PA guidelines, %	
Not aware	84.9%
Aware	15.1%
Knowledge about PA guidelines, %	
Inadequate	93.4%
Adequate	6.6%
Average score of items for belief about PA (range, 1 to 5), M (SD)	3.5 (0.8)
Average score of items for behavioral intention (range, 1 to 5), M (SD)	2.9 (1.1)
Volume of moderate-to-vigorous PA (METs-hours per day) measured by the JPHC Study’s questionnaire [31, 33], M (SD)	10.4 (12.4)
Activity level of PA measured by Kawakami et al. [32], %	
Low	69.0%
High	31.0%

*Abbreviations: JPHC study* Japan Public Health Center-based prospective study, *M* mean, *METS* metabolic equivalents, *PA* physical activity, *SD* standard deviation

### Pathways from PA guideline awareness to behavior among all respondents

**Fig. 2:**
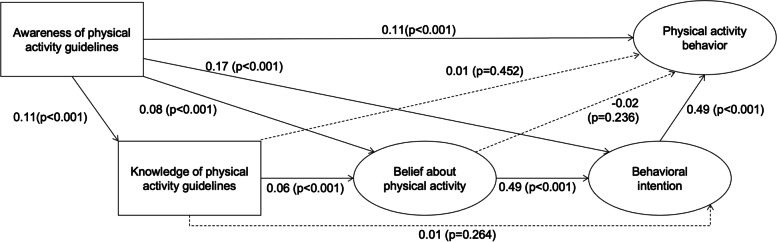
Initial model for the pathways from PA guideline awareness to behavior among all respondents. Solid lines indicate paths that were statistically significant, and dashed lines indicate paths that were not statistically significant. χ^2^(69) = 1425.9 (*p* < 0.001), GFI = 0.970, AGFI = 0.954, CFI = 0.975, TLI = 0.967, RMSEA = 0.053, AIC = 1497.8

**Fig. 3:**
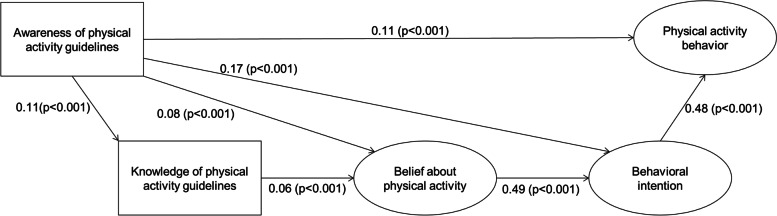
Revised structural model for PA pathways from guideline awareness to behavior among all respondents. Error/disturbance terms and observed variables for the items of latent variables were analyzed but not shown in this figure for readability purposes. The pathway coefficients were standardized. The direct, indirect, and total effects on PA behavior are shown in Table 2. χ^2^ (72) = 1429.2 (*p* < 0.001), CFI = 0.975, TLI = 0.969, RMSEA = 0.052, AIC = 1495.2

Figure 2 shows the initial model for the pathways from PA guideline awareness to behavior among all respondents based on the logic model. In the initial model, the path from knowledge to behavioral intention, from knowledge to PA behavior, and from belief to PA behavior were not statistically significant. These insignificant paths were removed from the revised model. Figure 3 shows the estimated standardized path coefficients in the revised model. The standardized total, direct, and indirect effects of the PA guidelines on its behavior in the revised model are indicated in Table 2. Table 2 shows that similar to the direct effect, the indirect and total effects of PA guideline awareness on its behavior, mediated by knowledge, belief, and behavioral intention, were statistically and positively significant. Thus, the findings supported the mediating roles of these psychological pathways.

**Table 2:** Standardized direct, indirect, and total effects in the revised structural model among all respondents

	Direct effect on PA behavior (95% CI)	*P*-value	Indirect effect on PA behavior (95% CI)	*P*-value	Total effect on PA behavior (95% CI)	*P*-value
Awareness of PA guidelines	0.11 (0.08, 0.14)	< 0.001	0.10 (0.08, 0.12)	< 0.001	0.21 (0.17, 0.25)	< 0.001
Knowledge of PA guidelines	―		0.01 (0.01, 0.02)	< 0.001	0.01 (0.01, 0.02)	< 0.001
Beliefs about PA	―		0.23 (0.21, 0.26)	< 0.001	0.23 (0.21, 0.26)	< 0.001
Behavioral intention	0.48 (0.43, 0.52)	< 0.001	―		0.48 (0.43, 0.52)	< 0.001

Direct, indirect, and total effects were standardized. The structural model is shown in Fig. 2

*CI* confidence interval, *PA* physical activity

### Moderating effects of health literacy on PA pathways from guideline awareness to behavior

The fit indices of the unconstrained model were CFI = 0.975, TLI = 0.969, RMSEA = 0.052, and AIC = 1550.8. The comparisons of fit indices between the unconstrained model and the models with equality constraints are shown in Table 3 for the main path coefficients among awareness, knowledge, belief, behavioral intention, and PA behavior and in Additional file 2 for other additional parameters. The changes in Chi-squared were significant for the paths from awareness to knowledge, awareness to belief, and awareness to PA behavior (Table 3). Thus, the moderating effects of health literacy on these psychological pathways were confirmed. Regarding the path from awareness to behavioral intention, knowledge to belief, belief to behavioral intention, and behavioral intention to PA behavior, the changes in Chi-squared were insignificant (Table 3). For other additional parameters, the changes in Chi-squared were significant as shown in Additional file 2. Regarding the final model of the multi-group structural equation modeling, the present study placed equality constraints for the path from awareness to behavioral intention, knowledge to belief, belief to behavioral intention, and behavioral intention to PA behavior.

**Table 3:** Fit indices of models with equality constraints of the main pathway coefficients

	χ^2^	Δχ^2^	*P*-values	CFI	TLI	RMSEA	AIC
Unconstrained	1418.8	―		0.973	0.966	0.036	1550.8
Equality constraint on path coefficient from awareness to knowledge	1441.4	22.7	< 0.001	0.973	0.966	0.036	1571.4
Equality constraint on path coefficient from awareness to belief	1429.9	11.2	0.001	0.973	0.966	0.036	1559.9
Equality constraint on path coefficient from awareness to behavioral intention	1420.4	1.7	0.199	0.973	0.966	0.035	1550.4
Equality constraint on path coefficient from awareness to PA behavior	1438.1	19.3	< 0.001	0.973	0.966	0.036	1568.1
Equality constraint on path coefficient from knowledge to belief	1421.2	2.5	0.117	0.973	0.966	0.035	1551.2
Equality constraint on path coefficient from belief to behavioral intention	1418.8	0.0	0.916	0.973	0.967	0.035	1548.8
Equality constraint on path coefficient from behavioral intention to PA behavior	1421.9	3.1	0.077	0.973	0.966	0.035	1551.9

Equality constraint was placed between the low- and high-health literacy groups

Δχ^2^, changes in Chi-squared; *AIC* Akaike's information criterion, *CFI* Comparative fit index, *PA* physical activity, *TLI* Tucker-Levis index, *RMSEA* root mean square error of approximation

**Fig. 4:**
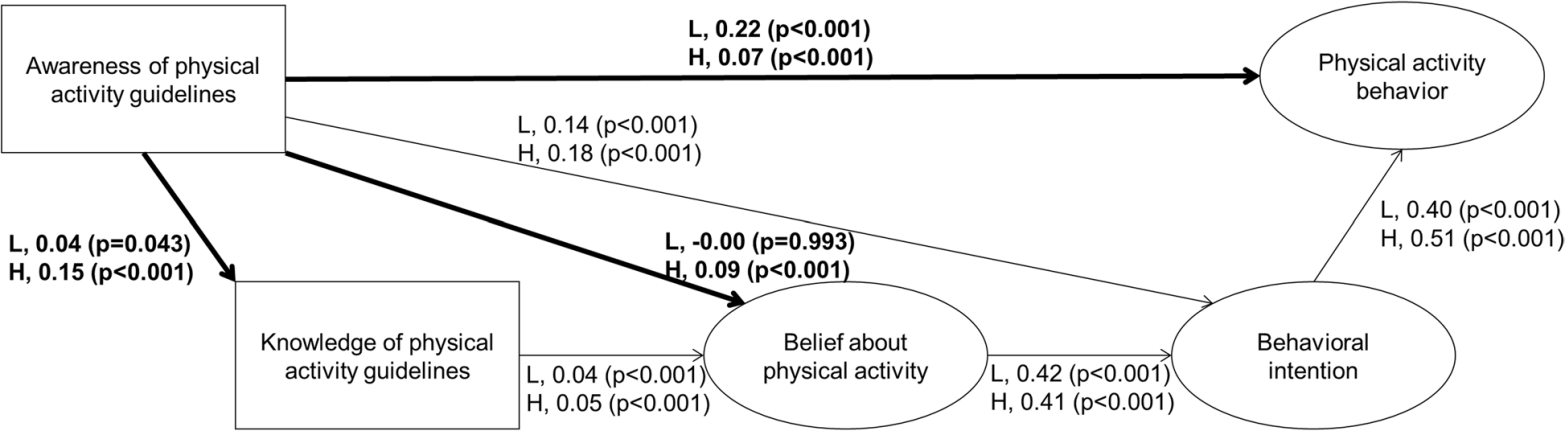
Multi-group structural model for PA pathways from guideline awareness to behavior in health literacy groups. The results of the high- and low-health literacy groups are presented (H, high-health literacy groups; L, low-health literacy groups). Bold and thin lines represent unconstrained and constrained pathways between high- and low-health literacy groups, respectively. The pathway coefficients were standardized. The direct, indirect, and total effects on PA behavior are shown in Table 4. χ^2^ (148) = 1425.9 (*p* < 0.001), CFI = 0.973, TLI = 0.967, RMSEA = 0.035, AIC = 1549.9

Figure 4 shows the final model of the multi-group structural equation modeling. While the path coefficients from awareness to knowledge were positive and statistically significant among both the low- and high-health literacy groups, the coefficient among the high-health literacy group was larger than that among the low-health literacy group. While the path coefficient from awareness to belief was positive and statistically significant among the high-health literacy group, it was not statistically significant among the low-health literacy group. The path coefficient from awareness to PA behavior among the low-health literacy group was larger than that among the high-health literacy group, while the coefficients were positive and statistically significant in both groups.

Direct, indirect, and total effects of PA guideline awareness on PA behavior in the final model among both groups are shown in Table 4. Among the low-health literacy group, the proportion of the direct effect of PA guideline awareness on PA behavior was larger than that of its indirect effects. In contrast, among the high-health literacy group, the proportion of the direct effect of the PA guideline awareness on PA behavior was smaller than that of its indirect effects.

**Table 4:** Standardized direct, indirect, and total effects in multi-group structural model stratified by health literacy

	Direct effect on PA behavior (95% CI)	*P*-value	Indirect effect on PA behavior (95% CI)	*P*-value	Total effect on PA behavior (95% CI)	*P*-value
Low health literacy group						
Awareness of PA guidelines	0.22 (0.15, 0.30)	< 0.001	0.06 (0.05, 0.07)	< 0.001	0.28 (0.21, 0.36)	< 0.001
Knowledge of PA guidelines	―		0.01 (0.00, 0.01)	< 0.001	0.01 (0.00, 0.01)	< 0.001
Beliefs about PA	―		0.17 (0.15, 0.20)	< 0.001	0.17 (0.15, 0.20)	< 0.001
Behavioral intention	0.40 (0.35, 0.46)	< 0.001	―		0.40 (0.35, 0.46)	< 0.001
High health literacy group						
Awareness of PA guidelines	0.07 (0.03, 0.11)	< 0.001	0.11 (0.10, 0.13)	< 0.001	0.18 (0.14, 0.23)	< 0.001
Knowledge of PA guidelines	―		0.01 (0.01, 0.02)	< 0.001	0.01 (0.01, 0.02)	< 0.001
Beliefs about PA	―		0.21 (0.19, 0.23)	< 0.001	0.21 (0.19, 0.23)	< 0.001
Behavioral intention	0.51 (0.46, 0.57)	< 0.001	―		0.51 (0.46, 0.57)	< 0.001

Direct, indirect, and total effects were standardized. The structural model is shown in Fig. 3

*CI* confidence interval, *PA* physical activity

## Discussion

The present study revealed that the awareness of PA guidelines is directly and indirectly associated with PA behavior through the mediation of knowledge, beliefs, and behavioral intentions. Furthermore, health literacy was related to the degree of association between direct and indirect pathways.

It is essential to examine the psychological pathway in Cavil & Bauman's logic model to explore strategies to promote behavior from the awareness of PA guidelines [11]. Many previous studies have examined associations between awareness and/or knowledge of PA guidelines and PA behavior [16, 18, 20–23, 29, 30]. However, the evidence about their associations is still unclear because some studies have reported null results [17, 24]. Furthermore, few previous studies have investigated the psychological pathways for their associations. To our best knowledge, this study is the first to reveal the psychological pathways, in which PA guideline awareness has a direct or indirect influence on PA behavior. Relevant studies on psychological pathways have reported that the awareness of mass media campaigns is directly or indirectly associated with PA behavior via the mediation of knowledge, beliefs, and behavioral intention [12–14]. Furthermore, a previous study for Japanese dietary guidelines also indicated a similar pathway from the awareness of dietary guidelines to eating behavior [15]. Therefore, taking these findings into account, the results of this study provide evidence to support Cavil & Bauman's logic model [11], which suggests that knowledge, beliefs, and behavioral intentions are mediating variables and important pathways in PA promotion for the awareness of PA guidelines. At the population level, PA behavior changes may require an extended time period to occur [10, 36, 37]. On the other hand, guideline awareness is categorized as a short-term outcome, and knowledge, beliefs, and behavioral intentions as medium-term outcomes [38]. In short, taking into account the results of this study, it is also helpful to assess awareness, knowledge, beliefs, and behavioral intentions as a pathway to PA behavior when conducting PA guideline-based interventions.

The Active Guide, summary sheet of Japanese physical activity guidelines, contains a lot of information to encourage PA behavior change, which may also be an important factor in these associations. In particular, the Active Guide is designed for ease of understanding through the use of illustrations to convey necessary information in a concise three-fold pamphlet format. As such, the appealing message of “Plus Ten” could be fully conveyed, and information that encourages environmental awareness could be included [8, 9]. This finding suggests that the awareness of the Active Guide is either directly or indirectly associated with PA behavior via knowledge, beliefs, and behavioral intentions. However, the rate of awareness of the Active Guide remains low, at approximately 15% [19, 23]. Therefore, further efforts to disseminate PA guidelines to the public need to be implemented.

Another important finding of this study is that the direct and indirect effects of the PA guideline awareness on PA behavior depend on health literacy. The high-health literacy group had a 0.63 times smaller proportion of the direct effect of awareness on PA behavior than that of the indirect effects. In contrast, the proportion of direct effect was 3.14 times larger in the low-health literacy group than in the high-health literacy group. Therefore, the high-health literacy group is more likely to translate PA guideline awareness into PA behavior indirectly and gradually via the psychological pathways of knowledge, beliefs, and behavioral intentions. In contrast, the low-health literacy group is more likely to translate PA guideline awareness into PA behavior directly without those pathways. Furthermore, our previous study [19] also showed that the higher the health literacy, the higher the awareness of the Active Guide and the higher the proportion of those with high MVPA and PA levels. A notable aspect of this previous study is that the positive association between awareness of the Active Guide and PA tended to be more pronounced in the low-health literacy group than in the high-health literacy group. This result suggests that awareness of the Active Guide promotes PA among people with low health literacy. In summary, in addition to improving health literacy for the population, it may be effective to consider PA guideline-based intervention methods to promote PA depending on the current health literacy level in the target population. In general, high health literacy is associated with high skills in obtaining health information. People with high health literacy are more likely to practice health behaviors than that observed in those with low health literacy [26]. In a systematic review, Buja et al. reported a positive association between high health literacy and high amounts of PA in 18 of 22 articles, emphasizing the importance of health literacy improvement [27]. Therefore, it is essential to implement initiatives to improve health literacy as a medium- to long-term perspective for promoting PA. In addition to this, when implementing PA interventions in high-health literacy populations, increasing PA guideline awareness as well as knowledge of and beliefs about PA may promote PA behavior change more efficiently. For example, it may be more effective for the high literacy group to include not only PA recommendations but also more advanced specialized information, such as knowledge of specific practices. On the other hand, when conducting PA interventions in low health literacy populations, it seems most important first to devise ways to increase awareness of PA guidelines. For instance, an approach, such as an Active Guide makes the information easy to understand and consistently appealing so that people can understand, evaluate, and use the content [19].

In the future, creating a strategy to increase awareness of the Active Guide is required in Japan. Milton et al. emphasized the need to conduct a stakeholder analysis and consider key audiences, communication aims, and approaches as examples of measures to maximize the effectiveness of PA guidelines [39]. However, De Cocker et al. [40] reported that the primary audience for PA recommendations had been health professionals, health practitioners, and policymakers. Few studies have developed and evaluated recommendation messages for the general public. Furthermore, as Williamson et al. [38] noted, more evidence regarding the contents and delivery methods for communicating PA messages is required. In particular, it was emphasized that developing PA recommendation messages aimed at the general public requires the involvement of the general public and experts [40]. In addition, future studies are needed to determine how the contents and delivery of messages should be devised, especially according to the level of health literacy, based in part on the findings of this study.

The strength of this study is its novel comprehensive assessment of awareness, knowledge, beliefs, and behavioral intentions regarding PA guidelines. The study also revealed the influence of health literacy on pathways to PA behavior. In addition, stratified random sampling was conducted among the registrants of a social survey company to match the distribution of sex, age, and educational background of the Japanese population. As such, this survey was conducted on a large scale with 7,000 participants. However, since stratified sampling was not conducted for current marital status, household income level, current working status, and PA level, their representativeness is not known. Furthermore, there are several limitations. First, this is a cross-sectional study; therefore, causality could not be established. Second, since this is an online survey of registrants of a social research firm, the possibility of sampling bias cannot be ruled out. Third, standardized assessment instruments for awareness, knowledge, beliefs and behavioral intentions in PA guidelines have yet to be established. Therefore, the probability of valid and reliable assessment instruments is needed in the future. In this study, the test–retest reliability of the beliefs section of the Active Guide is low (*r* = 0.45). Fourth, since this study is based on a logic model, we assumed that those with behavioral intentions would show more association with PA. Therefore, we did not assume here that participants were already physically active and did not have that intention. Fifth, the health literacy scale used in this study does not measure functional health literacy. Therefore, this study assumes that the participants have some degree of health literacy and that they do not have many problems with functional health literacy. Furthermore, the scale used in this study was divided into two groups based on median values since there is no cutoff point as of yet. However, this method is population-dependent, and results may differ between studies. Finally, we could not use accelerometers to objectively measure the amount of PA. To enhance the validity of these findings, a longitudinal study of the PA pathways from guideline awareness, with a reliable measurement of beliefs and objective PA measures, is warranted.

## Conclusions

This study revealed that in addition to the direct effect of PA guideline awareness on PA behavior, PA awareness has indirect effects on PA behavior, which are mediated by knowledge, beliefs, and behavioral intentions. Furthermore, for the high-health literacy group, the proportion of the direct effect of PA guideline awareness on PA behavior was lower than that of the indirect effects. In contrast, the direct effect compared to the indirect effect was higher in the low-health literacy group. In the future, when implementing PA guideline-based interventions or administrative programs, it is necessary to consider efforts to increase knowledge and beliefs as well as awareness of the guidelines for those with high health literacy. For those with low health literacy, efforts to improve awareness of the guidelines with easy-to-understand and appealing content may be necessary.

## Supplementary Information

**Additional file 1.** Results of a confirmatory factor analysis of beliefs about Active Guides. Confirmatory factor analysis revealed that the 10-item, one-factor model (model 1) had a poor model fit, the 8-item, one-factor model, with 2 items excluded, improved the goodness-of-fit index. Therefore, model 2 was adopted.

**Additional file 2.** Model fit indices of models with equality constraints of additional parameters between low- and high- health literacy groups. The comparisons of fit indices between the unconstrained model and the models with equality constraints are shown in Table 3 for the main path coefficients among awareness, knowledge, belief, behavioral intention, and PA behavior and in Additional file 2 for other additional parameters.

**Additional file 3.** STROBE Statement—checklist of items that should be included in reports of observational studies. Since this is an observational study, we have reported the article in accordance with the STROBE Statement.

## Data Availability

The datasets used and/or analyzed during the current study are available from the corresponding author on reasonable request.

## Abbreviations

AIC: Akaike's information criterion
CCHL: Communicative and Critical Health Literacy
CFI: Comparative fit index
JPHC study: Japan Public Health Center-based prospective study
MET: Metabolic equivalent
MVPA: Moderate-to-vigorous intensity physical activity
PA: Physical activity
RMSEA: Root mean square error of approximation
TLI: Tucker-Levis index
