# Supplementary material for: Does health literacy moderate the psychological pathways of physical activity from guideline awareness to behavior? A multi-group structural equation modeling
Source: BMC Public Health. 2023 Jan 14;23:106. doi: 10.1186/s12889-023-15012-3 (PMC9840824; doi:10.1186/s12889-023-15012-3)
Supplement: Supplementary file 1 — Additional file 1. Results of a confirmatory factor analysis of beliefs about Active Guides. Confirmatory factor analysis revealed that the 10-item, one-factor model (model 1) had a poor model fit, the 8-item, one-factor model, with 2 items excluded, improved the goodness-of-fit index. Therefore, model 2 was adopted. [file 12889_2023_15012_MOESM1_ESM.pdf]

Additional file 1. Results of a confirmatory factor analysis of beliefs about Active Guides.

	Model 1[a] standardized coefficients	Model 2[b] standardized coefficients
I think increasing the amount of time spent on physical activity, even if only a little, helps improve my health.	0.86	0.86
I think being physically active, such as walking and strength training, helps improve my health.	0.82	—
I think being physically active through vigorous housework (cleaning, washing clothes, etc.) helps improve my health.	0.82	0.82
I think being physically active through transportation, such as walking and cycling, helps improve my health.	0.75	0.84
I think being physically active by using the restroom further away or taking the stairs helps improve my health.	0.81	0.82
I think reflecting on my daily life helps me develop physical activity habits.	0.79	0.79
I think using the spare time, such as work and household chores, helps me develop physical activity habits.	0.84	0.85
I think becoming aware of opportunities and environments for physical activity around us helps me develop the physical activity habits.	0.86	0.87
I think reviewing my community and work environment helps me develop physical activity habits.	0.69	—
I think enjoying physical activity with family and friends helps me develop physical activity habits.	0.80	0.80

Each item was surveyed using a 5-point scale, ranging from 1 (completely disagree) to 5 (very strongly agree).

[a] $\chi 2(35) = 2594.1$ ($p<0.001$), RMSEA = 0.102, GFI = 0.914, AGFI = 0.865, CFI = 0.957, TLI = 0.945

[b] $\chi 2(16) = 704.0$ ($p<0.001$), RMSEA = 0.070, GFI = 0.974, AGFI = 0.953, CFI = 0.985, TLI = 0.979

Both Model 1 and Model 2 underwent confirmatory factor analysis with a one-factor model. Error correlations were not included in either model.

This table is an English translation of the table by Tajima et al (23).
