# Supplementary material for: Does health literacy moderate the psychological pathways of physical activity from guideline awareness to behavior? A multi-group structural equation modeling
Source: BMC Public Health. 2023 Jan 14;23:106. doi: 10.1186/s12889-023-15012-3 (PMC9840824; doi:10.1186/s12889-023-15012-3)
Supplement: Supplementary file 2 — Additional file 2. Model fit indices of models with equality constraints of additional parameters between low- and high- health literacy groups. The comparisons of fit indices between the unconstrained model and the models with equality constraints are shown in Table 3 for the main path coefficients among awareness, knowledge, belief, behavioral intention, and PA behavior and in Additional file 2 for other additional parameters. [file 12889_2023_15012_MOESM2_ESM.pdf]

Additional file 2. Model fit indices of models with equality constraints of additional parameters between low- and high-health literacy groups.

	χ^2	$\Delta\chi^2$	p values	CFI	TLI	RMSEA	AIC
Unconstrained	1418.8	—		0.973	0.966	0.036	1550.8
Equality constraints within beliefs about PA							
Path coefficients from latent to each observed variable	1553.8	135.0	<0.001	0.971	0.965	0.036	1671.8
Variances of each observed variable	1552.5	133.8	<0.001	0.971	0.965	0.036	1668.5
Equality constraints within behavioral intention							
Path coefficients from latent to each observed variable	1452.7	33.9	<0.001	0.973	0.966	0.036	1582.7
Variances of each observed variable	1465.8	47.0	<0.001	0.972	0.966	0.036	1593.8
Equality constraints within behavioral intention							
Path coefficients from latent to each observed variable	1425.7	7.0	0.008	0.973	0.966	0.036	1555.7
Variances of each observed variable	1459.1	40.4	<0.001	0.973	0.966	0.036	1587.1
Equality constraints of variances of main variables							
Variance of awareness	1509.3	90.5	<0.001	0.971	0.964	0.037	1639.3
Variance of knowledge	1504.1	85.3	<0.001	0.972	0.964	0.037	1634.1
Variance of belief	1434.9	16.2	<0.001	0.973	0.966	0.036	1564.9
Variance of behavioral intention	1424.7	5.9	0.015	0.973	0.966	0.036	1554.7
Variance of PA behavior	1424.2	5.4	0.020	0.973	0.966	0.036	1554.2

PA, Physical Activity; $\Delta\chi^{2,}$ Changes in Chi-square

Equality constraint was placed between the low- and high-health literacy groups.
