# Supplementary material for: Does health literacy moderate the psychological pathways of physical activity from guideline awareness to behavior? A multi-group structural equation modeling
Source: BMC Public Health. 2023 Jan 14;23:106. doi: 10.1186/s12889-023-15012-3 (PMC9840824; doi:10.1186/s12889-023-15012-3)
Supplement: Supplementary file 3 — Additional file 3. STROBE Statement—checklist of items that should be included in reports of observational studies. Since this is an observational study, we have reported the article in accordance with the STROBE Statement. [file 12889_2023_15012_MOESM3_ESM.pdf]

STROBE Statement—checklist of items that should be included in reports of observational studies

	Item No.	Recommendation	Page No.	Relevant text from manuscript
**Title and abstract**	1	(*a*) Indicate the study's design with a commonly used term in the title or the abstract	2	Abstract
		(*b*) Provide in the abstract an informative and balanced summary of what was done and what was found	2/3	Abstract
**Introduction**				
Background/rationale	2	Explain the scientific background and rationale for the investigation being reported	4-8	Background
Objectives	3	State specific objectives, including any prespecified hypotheses	8	Background
**Methods**				
Study design	4	Present key elements of study design early in the paper	8	Methods– Participants and data collection
Setting	5	Describe the setting, locations, and relevant dates, including periods of recruitment, exposure, follow-up, and data collection	8/9	Methods– Participants and data collection
Participants	6	(*a*) *Cohort study*—Give the eligibility criteria, and the sources and methods of selection of participants. Describe methods of follow-up<br>*Case-control study*—Give the eligibility criteria, and the sources and methods of case ascertainment and control selection. Give the rationale for the choice of cases and controls<br>*Cross-sectional study*—Give the eligibility criteria, and the sources and methods of selection of participants	8/9	Methods– Participants and data collection
		(*b*) *Cohort study*—For matched studies, give matching criteria and number of exposed and unexposed<br>*Case-control study*—For matched studies, give matching criteria and the number of controls per case	Not applicable	
Variables	7	Clearly define all outcomes, exposures, predictors, potential confounders, and effect modifiers. Give diagnostic criteria, if applicable	9-14	Methods– Measures
Data sources/ measurement	8*	For each variable of interest, give sources of data and details of methods of assessment (measurement). Describe comparability of assessment methods if there is more than one group	9-14	Methods– Measures
Bias	9	Describe any efforts to address potential sources of bias	8-14	Methods– Participants and data collection, Measures
Study size	10	Explain how the study size was arrived at	8/9	Methods– Participants and data collection

Continued on next page

Quantitative variables	11	Explain how quantitative variables were handled in the analyses. If applicable, describe which groupings were chosen and why	9-14	Methods– Measures
Statistical methods	12	(*a*) Describe all statistical methods, including those used to control for confounding	14-17	Methods– Statistical analysis
		(*b*) Describe any methods used to examine subgroups and interactions	15-17	Methods– Statistical analysis
		(*c*) Explain how missing data were addressed	Not applicable	
		(*d*) *Cohort study*—If applicable, explain how loss to follow-up was addressed<br>*Case-control study*—If applicable, explain how matching of cases and controls was addressed<br>*Cross-sectional study*—If applicable, describe analytical methods taking account of sampling strategy	Not applicable	
		(*e*) Describe any sensitivity analyses	15/16	Methods– Statistical analysis
**Results**				
Participants	13*	(a) Report numbers of individuals at each stage of study—eg numbers potentially eligible, examined for eligibility, confirmed eligible, included in the study, completing follow-up, and analysed	8/9	Methods– Participants and data collection
		(b) Give reasons for non-participation at each stage	Not applicable	
		(c) Consider use of a flow diagram	Not applicable	
Descriptive data	14*	(a) Give characteristics of study participants (eg demographic, clinical, social) and information on exposures and potential confounders	17/18	Results– Participant characteristics, Table 1
		(b) Indicate number of participants with missing data for each variable of interest	Not applicable	
		(c) *Cohort study*—Summarise follow-up time (eg, average and total amount)	Not applicable	
Outcome data	15*	*Cohort study*—Report numbers of outcome events or summary measures over time	Not applicable	
		Case-control study—Report numbers in each exposure category, or summary measures of exposure	Not applicable	
		Cross-sectional study—Report numbers of outcome events or summary measures	18-20	Results– Pathways from awareness of the PA guidelines to physical activity behavior among all respondents, Moderating effects of health literacy on pathways from the awareness of PA guidelines to PA Behavior, Table2-4, Figure 2/3

Main results	16	(*a*) Give unadjusted estimates and, if applicable, confounder-adjusted estimates and their precision (eg, 95% confidence interval). Make clear which confounders were adjusted for and why they were included	18-20	Results– Pathways from awareness of the PA guidelines to physical activity behavior among all respondents, Moderating effects of health literacy on pathways from the awareness of PA guidelines to PA Behavior, Table2-4, Figure 2/3
		(*b*) Report category boundaries when continuous variables were categorized	Not applicable	
		(*c*) If relevant, consider translating estimates of relative risk into absolute risk for a meaningful time period	Not applicable	

Continued on next page

Other analyses	17	Report other analyses done—eg analyses of subgroups and interactions, and sensitivity analyses	18-20	Results– Pathways from awareness of the PA guidelines to physical activity behavior among all respondents, Moderating effects of health literacy on pathways from the awareness of PA guidelines to PA Behavior, Table3/4, Figure 3
**Discussion**				
Key results	18	Summarise key results with reference to study objectives	21	Discussion
Limitations	19	Discuss limitations of the study, taking into account sources of potential bias or imprecision. Discuss both direction and magnitude of any potential bias	25/26	Discussion
Interpretation	20	Give a cautious overall interpretation of results considering objectives, limitations, multiplicity of analyses, results from similar studies, and other relevant evidence	21-24	Discussion
Generalisability	21	Discuss the generalisability (external validity) of the study results	25	Discussion
**Other information**				
Funding	22	Give the source of funding and the role of the funders for the present study and, if applicable, for the original study on which the present article is based	28	Funding

*Give information separately for cases and controls in case-control studies and, if applicable, for exposed and unexposed groups in cohort and cross-sectional studies.

Note: An Explanation and Elaboration article discusses each checklist item and gives methodological background and published examples of transparent reporting. The STROBE checklist is best used in conjunction with this article (freely available on the Web sites of PLoS Medicine at http://www.plosmedicine.org/, Annals of Internal Medicine at http://www.annals.org/, and Epidemiology at http://www.epidem.com/). Information on the STROBE Initiative is available at www.strobe-statement.org.
